# A Mouse Model of Post-Arthroplasty *Staphylococcus aureus* Joint Infection to Evaluate *In Vivo* the Efficacy of Antimicrobial Implant Coatings

**DOI:** 10.1371/journal.pone.0012580

**Published:** 2010-09-07

**Authors:** Nicholas M. Bernthal, Alexandra I. Stavrakis, Fabrizio Billi, John S. Cho, Thomas J. Kremen, Scott I. Simon, Ambrose L. Cheung, Gerald A. Finerman, Jay R. Lieberman, John S. Adams, Lloyd S. Miller

**Affiliations:** 1 Orthopaedic Hospital Research Center, Orthopaedic Hospital Department of Orthopaedic Surgery, David Geffen School of Medicine at University of California Los Angeles, Los Angeles, California, United States of America; 2 Division of Dermatology, Department of Medicine, David Geffen School of Medicine at University of California Los Angeles, Los Angeles, California, United States of America; 3 Department of Biomedical Engineering, University of California Davis, Davis, California, United States of America; 4 Department of Microbiology and Immunology, Dartmouth Medical School, Hanover, New Hampshire, United States of America; 5 New England Musculoskeletal Institute, Department of Orthopaedic Surgery, University of Connecticut Health Center, Farmington, Connecticut, United States of America; Columbia University, United States of America

## Abstract

**Background:**

Post-arthroplasty infections represent a devastating complication of total joint replacement surgery, resulting in multiple reoperations, prolonged antibiotic use, extended disability and worse clinical outcomes. As the number of arthroplasties in the U.S. will exceed 3.8 million surgeries per year by 2030, the number of post-arthroplasty infections is projected to increase to over 266,000 infections annually. The treatment of these infections will exhaust healthcare resources and dramatically increase medical costs.

**Methodology/Principal Findings:**

To evaluate novel preventative therapeutic strategies against post-arthroplasty infections, a mouse model was developed in which a bioluminescent *Staphylococcus aureus* strain was inoculated into a knee joint containing an orthopaedic implant and advanced *in vivo* imaging was used to measure the bacterial burden in real-time. Mice inoculated with 5×10^3^ and 5×10^4^ CFUs developed increased bacterial counts with marked swelling of the affected leg, consistent with an acute joint infection. In contrast, mice inoculated with 5×10^2^ CFUs developed a low-grade infection, resembling a more chronic infection. *Ex vivo* bacterial counts highly correlated with *in vivo* bioluminescence signals and EGFP-neutrophil fluorescence of LysEGFP mice was used to measure the infection-induced inflammation. Furthermore, biofilm formation on the implants was visualized at 7 and 14 postoperative days by variable-pressure scanning electron microscopy (VP-SEM). Using this model, a minocycline/rifampin-impregnated bioresorbable polymer implant coating was effective in reducing the infection, decreasing inflammation and preventing biofilm formation.

**Conclusions/Significance:**

Taken together, this mouse model may represent an alternative pre-clinical screening tool to evaluate novel *in vivo* therapeutic strategies before studies in larger animals and in human subjects. Furthermore, the antibiotic-polymer implant coating evaluated in this study was clinically effective, suggesting the potential for this strategy as a therapeutic intervention to combat post-arthroplasty infections.

## Introduction

The incidence of infections after total joint replacement surgery has increased over the past decade despite the widespread use of intravenous antibiotic prophylaxis and a focus on aseptic surgical technique [1], [2]. Post-arthroplasty infections still occur in ∼1.2% of primary arthroplasties and 3–5% of revisions [1], [3]–[5]. As the demand for joint replacements increases with the aging population, the total number of infections is projected to rise from 17,000 to 266,000 per year by 2030 as the number of arthroplasties exceeds 3.8 million surgeries [1], [2], [6], [7]. The treatment of a post-arthroplasty infection is exceedingly difficult. Bacteria (especially S. aureus) form extracellular anionic polysaccharide biofilms on implanted metallic/plastic materials that block penetration of immune cells and antibiotics, promoting bacterial survival [8]–[11]. Once a biofilm is formed, surgical removal of all the implanted materials is necessary [8]–[11]. Most of these infections are caused by staphylococcal species (∼70%) [12]–[14] and an increasing number are due to virulent antibiotic-resistant strains such as methicillin-resistant S. aureus (MRSA) [15], which further complicate treatment [12]–[14].

The current standard of care in the U.S. to treat a chronic post-arthroplasty infection is a two-stage procedure beginning with (1) surgical removal of all prosthetic components and bone cement, debridement of necrotic/granulation tissue, placement of an antibiotic-impregnated spacer, administration of a 6-week course of intravenous antibiotics (during which the patient is unable to bear weight on the affected limb), and (2) revision arthroplasty after the infection has cleared [16]–[21]. In severe infections and refractory cases, arthrodesis, resection arthroplasty and amputation are sometimes necessary [22]–[24]. In the elderly, these infections result in increased mortality [25], [26]. Overall, the treatment of post-arthroplasty infection involves extensive medical and surgical care, prolonged disability/rehabilitation and significantly worse outcomes [1], [2], [7]. In addition, these infections represent an enormous economic burden due to additional medical costs and resource utilization as well as indirectly through lost wages and productivity [1], [2], [6]. These medical costs alone average $144,514 (compared with $30,173 for an uncomplicated arthroplasty) [2], which correspond to an annual national healthcare burden of $8.63 billion by 2015 [7].

Most post-arthroplasty infections are thought to be caused by invading bacteria at the time of surgery [27]–[29]. As treatment of infected implanted materials is exceedingly difficult, especially due to the inherent difficulties in treating an established biofilm [30], [31], one potential therapeutic strategy is to focus on the prevention of infection [27]–[29]. Previous animal models used to study post-arthroplasty joint infection have been performed in dogs [32]–[34], rabbits [35]–[41] and rats [42], [43]. These studies suggested that local antibiotic therapy in the joint at the time of surgery, including direct antibiotic or antimicrobial agents that are coated or covalently-linked directly to the prosthetic materials, can decrease post-arthroplasty infections by preventing bacterial seeding of the implants at the time of surgery [32]–[43]. Although these studies using larger animals provide extremely useful preclinical information, these studies are costly, labor intensive and require significant animal usage as euthanasia is required to determine the bacteria burden. The major goal of this study was to develop a mouse model of post-arthroplasty *Staphylococcus aureus* infection that would combine the use of bioluminescent bacteria and genetically engineered mice that possess fluorescent neutrophils (LysEGFP mice) with advanced techniques of *in vivo* whole animal imaging to noninvasively measure infection and inflammation in real-time, without requiring euthanasia. It is our hope that this model could be used as a rapid, accurate and inexpensive *in vivo* preclinical screening tool to evaluate the *in vivo* efficacy of potential strategies to prevent or treat post-arthroplasty infections. Therapeutic strategies that were successful in this mouse model could be confirmed in more extensive large animal or human studies, preventing the need to perform these expensive and time consuming studies on all candidate therapies.

## Materials And Methods

### Ethics statement

All animals were handled in strict accordance with good animal practice as defined in the federal regulations as set forth in the Animal Welfare Act (AWA), the 1996 Guide for the Care and Use of Laboratory Animals, PHS Policy for the Humane Care and Use of Laboratory Animals, as well as UCLA's policies and procedures as set forth in the UCLA Animal Care and Use Training Manual, and all animal work was approved by the UCLA Chancellor's Animal Research Committee (ARC#: 2008-112).

### *S. aureus* bioluminescent strain

The bioluminescent *S. aureus* SH1000 strain, ALC2906, which contains the shuttle plasmid pSK236 with the penicillin-binding protein 2 (*pbp2*) promoter fused to the *luxABCDE* reporter cassette from *Photorhabdus luminescens*, was used in all experiments [44]–[46]. This *S. aureus* strain naturally emits bioluminescent signals from live, actively metabolizing bacteria in all stages of the *S. aureus* life cycle [44]–[46].

### Preparation of *S. aureus* for inoculation into the joint space

*S. aureus* bioluminescent strain ALC2906 has a chloramphenicol resistance selection marker and chloramphenicol (10 µg/ml; Sigma-Aldrich) was supplemented to all cultures. *S. aureus* was streaked onto tryptic soy agar plates (tryptic soy broth [TSB] plus 1.5% bacto agar [BD Biosciences]) and grown at 37°C overnight [44]–[46]. Single colonies of *S. aureus* were cultured in TSB and grown overnight at 37°C in a shaking incubator (240 rpm) (MaxQ 4450; Thermo) [44]–[46]. Mid-logarithmic phase bacteria were obtained after a 2 h subculture of a 1/50 dilution of the overnight culture [44]–[46]. Bacterial cells were pelleted, resuspended and washed 3x in PBS. Bacterial concentrations were estimated by measuring the absorbance at 600 nm (A_600_; Biomate 3 [Thermo]). Colony forming units (CFUs) were verified after overnight culture of plates [44]–[46].

### Mice

12-week old male C57BL/6 wildtype mice were used (Jackson Laboratories). In some experiments, 12-week old male LysEGFP mice, a genetically engineered mouse line on a C57BL/6 background possessing green-fluorescent myeloid cells (mostly neutrophils) as a consequence of ‘knockin’ of enhanced green fluorescence protein (EGFP) into the lysozyme M gene, were used [47], [48].

### Mouse surgical procedures

**Figure 1:**
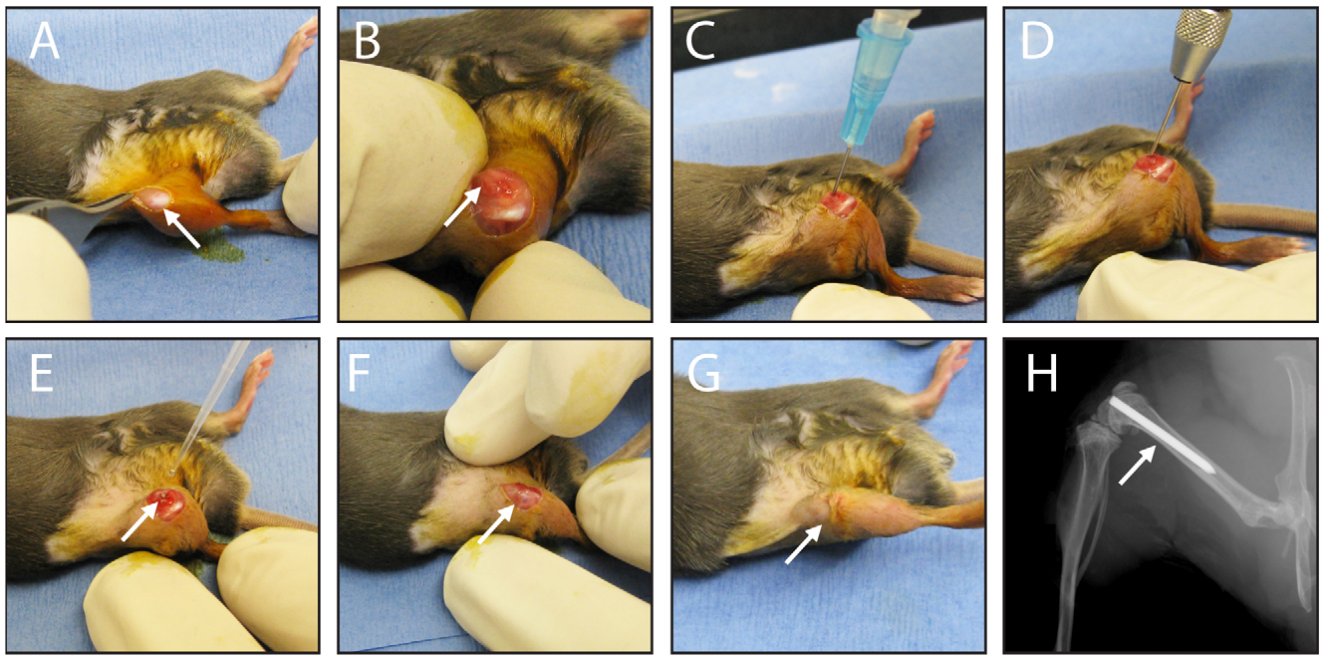
Mouse surgical procedures. (A) An incision was made in the skin overlying the right knee joint (arrow). (B) A medial parapatellar arthrotomy with lateral displacement of the quadriceps-patellar complex was performed to locate the intercondylar femoral notch (arrow). (C) An intramedullary canal was manually reamed into the distal femur with a 25 gauge needle. (D) An orthopaedic-grade stainless steel K-wire (diameter 0.6 mm) was surgically placed in a retrograde fashion into the intramedullary canal and cut so that the cut end extended 1 mm into the joint space. (E) An inoculum of *S. aureus* in a 2 µl volume was pipetted into the joint space (arrow). (F) The quadriceps-patellar complex was reduced back to the midline (arrow) and (G) the surgical site was closed with subcutaneous 5-0 Dexon sutures (arrow). (H) A representative radiographic image demonstrating the placement of the implant in the femoral canal with the cut end extending into the knee joint.

Mice were anesthetized via inhalation isoflurane (2%). The surgical procedure was modified from previous work [49], [50]. A skin incision was made over the right knee (Figure 1A). The distal right femur was accessed through a medial parapatellar arthrotomy with lateral displacement of the quadriceps-patellar complex (Figure 1B). After locating the femoral intercondylar notch (Figure 1B), the femoral intramedullary canal was manually reamed with a 25 gauge needle (Figure 1C). An orthopaedic-grade stainless steel Kirschner (K)-wire (diameter 0.6 mm) (Synthes) was surgically placed in a retrograde fashion and cut with 1 mm protruding into the joint space (Figure 1D). An inoculum of *S. aureus* in 2 µl of normal saline was pipetted into the joint space containing the cut end of the implant (Figure 1E). The quadriceps-patellar complex was reduced to the midline (Figure 1F) and the surgical site was closed with Dexon 5-0 sutures (Figure 1G). A representative radiograph demonstrates the position of the implant with good intramedually fixation of the stem and prominence of the cut surface in the joint (Figure 1H). Buprenorphine (0.1 mg/kg) was administered subcutaneously every 12 hours as an analgesic for the duration of the experiment.

### Quantification of *in vivo S. aureus* (*in vivo* bioluminescence imaging and colony forming units [CFUs])

Mice were anesthetized via inhalation of isoflurane (2%) and *in vivo* bioluminescence imaging was performed by using the Xenogen *in vivo* imaging system (Xenogen IVIS®; Caliper Life Sciences) [44]–[46]. Data are presented on color scale overlaid on a grayscale photograph of mice and quantified as maximum flux (photons per second (s) per cm^2^ per steradian (sr) [p/s/cm^2^/sr]) within a circular region of interest (1×10^3^ pixels) by using Living Image® software (Xenogen). To confirm that the bioluminescence signals corresponded to the bacterial burden *in vivo*, bacteria adherent to the implants were quantified by detaching the bacteria from the implant by sonication in 1 ml 0.3% Tween-80 in TSB for 10 minutes followed by vortexing for 5 minutes as previously described [51]. In addition, bacteria in the joint tissue were confirmed by homogenizing bone and joint tissue (Pro200® Series homogenizer; Pro Scientific). The number of bacterial CFUs that were adherent to the implant and in the joint tissue was determined by counting CFUs after overnight culture of plates and was expressed as total CFUs harvested from the implant and joint tissue.

### Quantification of neutrophil recruitment to the infected post-operative joint (*in vivo* fluorescence imaging)

To obtain a measurement of neutrophil infiltration, LysEGFP mice were used. After *in vivo* bioluminescence imaging, *in vivo* fluorescence imaging was performed by using the Xenogen IVIS® (Caliper Life Sciences). EGFP-expressing neutrophils within the post-operative site were visualized by using the GFP filter for excitation (445–490 nm) and emission (515–575 nm) at an exposure time of 0.5 seconds [47], [48]. Data are presented on color scale overlaid on a grayscale photograph of mice and quantified as total flux (photons/s) within a circular region of interest (1×10^3^ pixels) by using Living Image® software (Xenogen).

### Histologic analysis

Mice were euthanized via inhalation carbon dioxide and joint specimens were fixed in formalin (10%) overnight. Specimens were decalcified by incubation in Decalcifier II® solution (Surgipath) for 6 h and specimens were processed and embedded in paraffin. Sagittal sections of 4 µm thickness were cut and then were stained with hematoxylin and eosin (H&E) and Gram stain.

### Variable-pressure scanning electron microscopy

A field emission variable pressure scanning electron microscope (FE-SEM Zeiss Supra VP40) was used to obtain a digital image of the cut end of the implants. Conductive graphite glue was used to position the pins on a graphite stub. Pressure in the microscope chamber was maintained at 25Pa, which allowed the examination of the implant surface without the need of sputter coating. Secondary and in-lens detectors were used to reveal the topographical characteristics of the surface. Examination of the implant occurred at regular intervals by tilting the pin between −4 and 10 degrees and rotating it every 30 degrees for a total of 360 degrees.

### Coating of metallic implants with an antibiotic-impregnated bioresorbable polymer

A bioresorbable polymer impregnated with rifampin and minocycline, which was modified from FDA-approved mesh coatings to prevent infection of pacemakers and implantable cardioverter defibrillators (Pivit™ AB and AigisRx™ CRM; TyRx Pharma, Inc.), was used (50). To coat the stainless steel K-wire implants with this antibiotic-impregnated polymer, K-wires were hand-dipped in a mixture of a bioresorbable tyrosine-derived polyesteramide rifampin and minocycline and methylene chloride. Vehicle coating consisted of bioresorbable tyrosine-derived polyesteramide and methylene chloride only (no antibiotic). The coated pins were heat dried for at least 12 h until residual solvent was less than 600 ppm, stored at −15°C and sterilized by gamma irradiation. Three different formulations were generated (Coatings A, B and C) with the following approximate antibiotic concentrations: Coating A: 32.5 µg/mm^3^ of rifampin and 36.1 µg/mm^3^ of minocycline; Coating B: 46.1 µg/mm^3^ of rifampin and 47.7 µg/mm^3^ of minocycline; and Coating C: 97.4 µg/mm^3^ of rifampin and 104.2 µg/mm^3^ of minocycline. The coatings were repeatedly dipped until the thickness of Coating A and Coating B were ∼40–45 µm whereas and Coating C was ∼80–90 µm. Thus, Coatings A and B would elute at the same rate whereas Coating C would elute slower because it had double the coating thickness.

### Statistical analysis

Data were compared by using a Student's *t*-test (two-tailed). All data are expressed as mean ± standard error of the mean (sem) where indicated. Values of *p*<0.05, *p*<0.01 and *p*<0.001 were considered statistically significant.

## Results

### *In vivo* bioluminescence imaging to measure the bacterial burden in real-time

To model a post-arthroplasty infection, an orthopaedic-grade K-wire (Synthes, Inc., West Chester, PA) was surgically placed into the femur with the cut end protruding into knee joint and an inoculum of *S. aureus* was pipetted into the joint space before closure (Figure 1). To measure the bacterial burden within the infected post-operative joints in real-time, we used a bioluminescent *S. aureus* strain (SH1000) that naturally emits lights from live, ATP-producing bacteria at all stages of the *S. aureus* life cycle [44]–[46]. The bacterial burden was subsequently measured on post-operative days 0, 1, 3, 5, 7 and 10 in anesthetized mice in real-time by using the Xenogen *in vivo* imaging system (Xenogen IVIS®; Caliper Life Sciences) [44]–[46].

**Figure 2:**
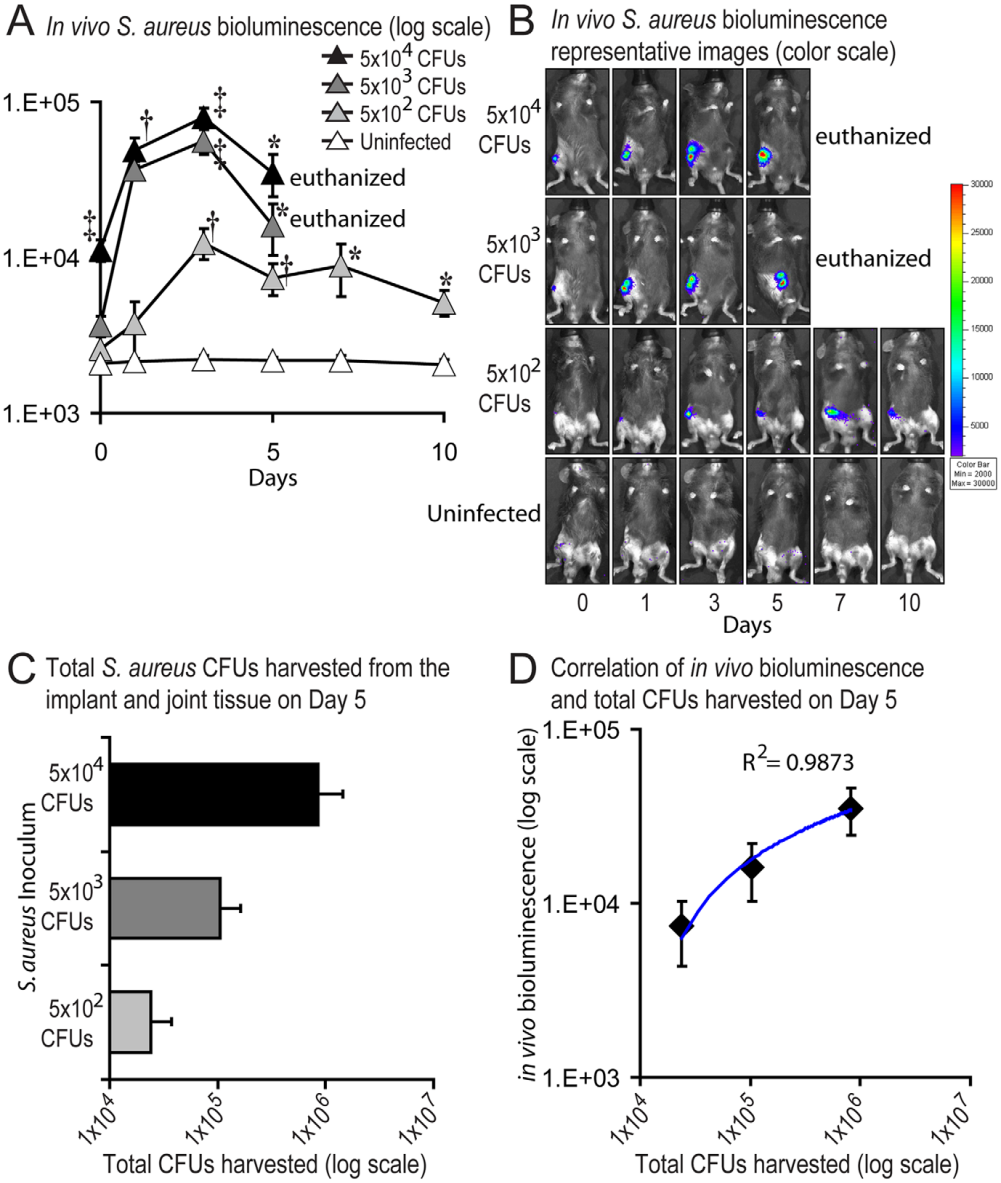
Measurement of bacterial burden using *in vivo* bioluminescence. After surgical placement of an orthopaedic-grade stainless steel K-wire into the distal femur, 5×10^4^, 5×10^3^ and 5×10^2^ CFUs/2 µl of *S. aureus* or 2 µl of saline alone (uninfected) were inoculated into the knee joint tissue in the area of the cut end of the implant (n = 7 mice per group). (A) Bacterial counts as measured by *in vivo S. aureus* bioluminescence (mean maximum flux [p/s/cm^2^/sr] ± sem) (logarithmic scale). (B) Representative *in vivo S. aureus* bioluminescence on a color scale overlaid on top of a grayscale image of mice. (C) Bacteria adherent to the implants and present in the joint tissue were harvested from mice on post-operative day 5 and CFUs were determined after overnight culture. (D) Correlation between *in vivo* bioluminescence signals and total CFUs harvested from the infected implants and joint tissue on post-operative day 5. The logarithmic trendline (blue line) and the correlation coefficient of determination (R^2^) between *in vivo* bioluminescence signals and total CFUs are shown. Data are expressed as mean CFUs ± sem. *p<0.05 †*p*<0.01 *S. aureus* inoculated mice versus uninfected mice (Student's t-test).

To determine the optimal bacterial inoculum to produce a chronic implant infection, C57BL/6 mice were inoculated with increasing logarithmic concentrations of *S. aureus* (5×10^2^, 5×10^3^ and 5×10^4^ CFUs/2 µl). During the first 5 days after the inoculation, mice that received 5×10^3^ or 5×10^4^ CFUs had 20- to 50-fold higher bioluminescence signals than uninfected mice (Figure 2A, B). Clinically, both of these groups of mice developed marked inflammation as characterized by increased swelling and decreased mobility of the affected leg and were euthanized on post-operative day 5. Thus, inocula of 5×10^3^ or 5×10^4^ CFUs of *S. aureus* induced markedly high bioluminescent signals and produced clinical signs of infection that was consistent with an acute purulent joint infection. In contrast, mice that received an inoculum of 5×10^2^ CFUs developed signs of infection in the affected leg that were only minimally different than uninfected mice. These mice had up to 6- to 8-fold higher bioluminescence signals than the background levels of uninfected control mice at all post-operative days through day 10 (Figure 2A, B). The mild clinical findings combined with the low level of bacterial bioluminescence allowed us to follow the infection in the mice that received the inoculum of 5×10^2^ CFUs for at least 10 days, which more closely resembled a chronic and persistent infection. Thus, the inoculum of 5×10^2^ CFUs was used in all subsequent experiments.

To confirm that the *in vivo* bioluminescence signals accurately represented the bacterial burden *in vivo*, traditional bacterial counts were performed on post-operative day 5 from bacteria adherent to the implant and present in the joint tissue (Figure 2C). Mice that were inoculated with 5×10^4^, 5×10^3^ and 5×10^2^ CFUs had a total bacterial burden *ex vivo* of 8.3×10^5^, 1×10^5^ and 2.4×10^4^ CFUs, respectively (Figure 2C). In addition, the *in vivo* bioluminescent signals correlated with the corresponding *ex vivo* bacterial CFUs (correlation coefficient of determination: R^2^ = 0.9873; Figure 2D), suggesting that the *in vivo* bioluminescence signals at least through day 5 provided an approximation of the actual bacterial burden *in vivo*. However, since the bacterial strain used had the lux genes in a plasmid that is maintained *in vitro* under chloramphenicol selection, the plasmid is likely lost during the *in vivo* infection over time. In broth culture without selection, the plasmid was stable for the first 3 days *in vitro* with greater than 97% of bacteria still containing the plasmid whereas only 53%, 38% and 21% of the bacteria still contained the plasmid on days 5, 7 and 10, respectively (data not shown). Thus, although the bioluminescent signals obtained with this strain provide an approximation of the bacterial burden *in vivo*, it is likely an underestimate of the actual bacterial burden, especially at later time points.

### *In vivo* fluorescence imaging to measure neutrophil infiltration in real-time

**Figure 3:**
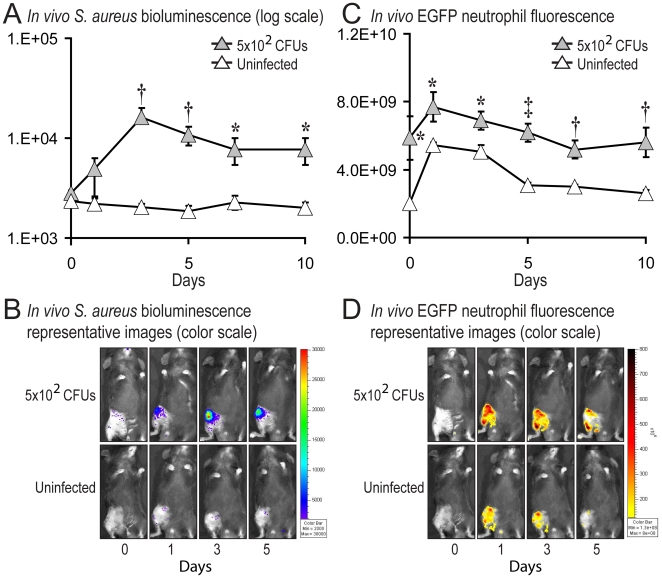
Measurement of bacterial burden and neutrophil infiltration using *in vivo* bioluminescence and fluorescence imaging. After surgical placement of an orthopaedic-grade stainless steel K-wire into the distal femur, 5×10^2^ CFUs/2 µl of *S. aureus* or 2 µl of saline alone (uninfected) was inoculated into the knee joint in the area of the cut end of the implant (n = 6 mice per group). (A) Bacterial counts as measured by *in vivo S. aureus* bioluminescence (mean maximum flux [p/s/cm^2^/sr] ± sem) (logarithmic scale). (B) Representative *in vivo* bioluminescence on a color scale overlaid on top of a grayscale image of mice. (C) Neutrophil infiltration (EGFP neutrophil fluorescence) as measured *by in vivo* fluorescence (total flux [photons/sec] ± sem). (D) Representative neutrophil infiltration as measured by a color scale of fluorescence overlaid on top of a grayscale image of mice. *p<0.05 †*p*<0.01 ‡*p*<0.001 *S. aureus* inoculated mice versus uninfected mice (Student's t-test).

The degree of inflammation within the post-operative knee joints was measured by quantifying neutrophil infiltration, a key correlate for inflammation and infection. This was accomplished by using *in vivo* fluorescence imaging of LysEGFP mice, a genetically engineered mouse strain that possesses green-fluorescent neutrophils [47], [48]. The bioluminescent *S. aureus* strain infected into the knee joints of LysEGFP mice enabled simultaneous measurement of both bacterial burden and neutrophil infiltration on post-operative days 0, 1, 3, 5, 7 and 10 (Figure 3). Similar to C57BL/6 mice in Figure 2, *S. aureus* (5×10^2^ CFUs)-infected LysEGFP mice developed bioluminescence signals that were up to 8-fold higher than the background levels of uninfected control mice through day 10 (Figure 3A). In addition, the *S. aureus*-infected LysEGFP mice had 20–40% higher EGFP-neutrophil fluorescent signals than uninfected control mice on all post-operative days 1 to 10 (Figure 3B). This degree of neutrophil recruitment, confirms our clinical observations that the inoculum of 5×10^2^ CFUs produced a low-grade inflammatory response, suggesting that EGFP-neutrophil fluorescence provides a quantifiable measurement of the clinical inflammation observed in our model.

### Histologic analysis of post-operative knee joints

**Figure 4:**
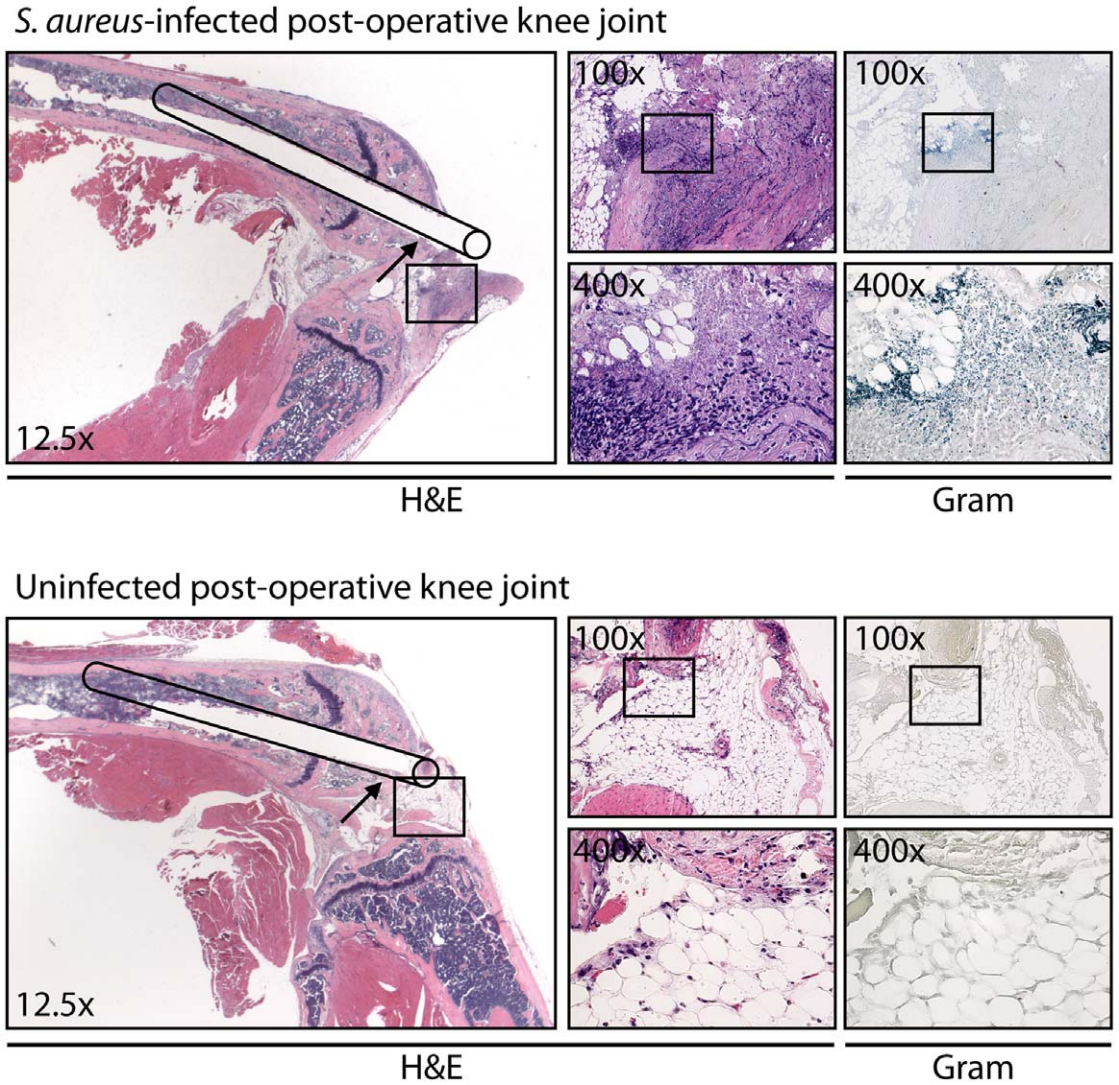
Histologic analysis. 5×10^2^ CFUs/2 µl of *S. aureus* or 2 µl of saline alone (uninfected) was inoculated into the knee joint in the area of the cut end of the implant. At post-operative day 1, the implants were removed and the joint tissue was fixed in formalin, decalcified and embedded in paraffin. Sagittal sections (4 µm) of the joint tissue were subsequently stained with hematoxylin and eosin (H&E) and Gram stain. Representative photomicrographs of histologic sections are shown (1 of 3 mice per group, with similar results). Left large panels: low magnification (12.5x) of H&E stained joint specimens with a line drawing of the location of the implant with the intramedullary canal seen within the femur. Upper right small panels: higher magnification (100x) of H&E- and Gram-stained joint specimens of the boxed area in the left panel at the location of the cut end of the implant within the joint. Lower right small panels: higher magnification (400x) of H&E- and Gram-stained section in the boxed areas in the upper right panels.

To determine the location of the inflammatory infiltrate and bacterial inoculum within the infected post-operative joints, histologic sections were harvested from *S. aureus*-inoculated (5×10^2^ CFUs) and uninfected control mice on post-operative day 1 (Figure 4). Mice inoculated with *S. aureus* had increased neutrophils in the joint tissue as seen in hematoxylin & eosin (H&E) stained sections. In addition, Gram-positive (blue-staining) bacteria could be readily detected in areas of inflammatory cells. In contrast, uninfected control mice that only had the surgical implant placed had minimal neutrophil infiltration and no bacteria were detected by Gram-stain. These histologic findings corroborate our *in vivo* bioluminescence and fluorescence imaging data demonstrating that the inoculum of 5×10^2^ CFUs of *S. aureus* induced neutrophil infiltration and bacterial proliferation in the joint tissue in the area of the implant.

### Detection of biofilm formation on the metallic implants

**Figure 5:**
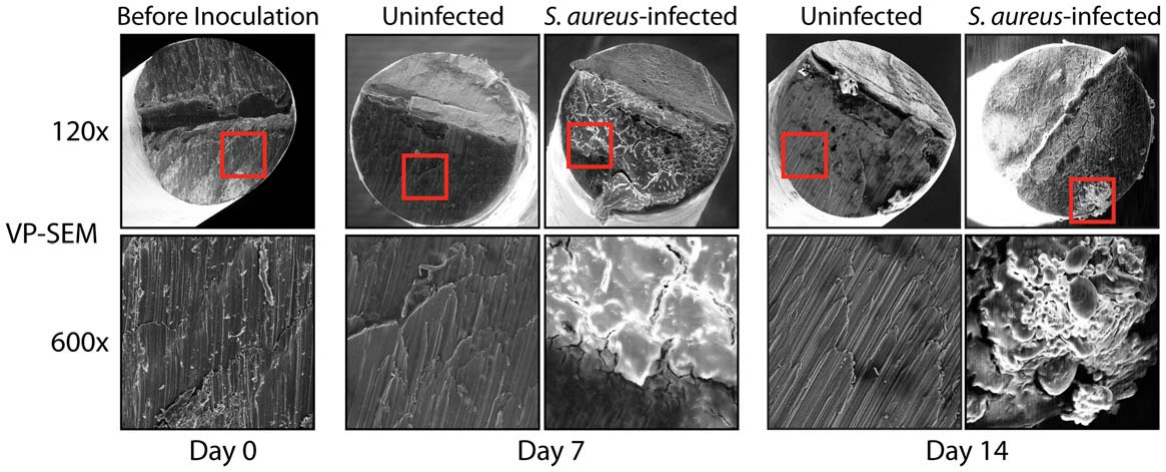
Biofilm formation on the implant as visualized by variable-pressure scanning electron microscopy (VP-SEM). 5×10^2^ CFUs/2 µl of *S. aureus* or 2 µl of saline alone (uninfected) was inoculated into the knee joint in the area of the cut end of the implant. At post-operative days 0 (before inoculation), 7 and 14, the implants were harvested and the cut ends of the implants that were in the joint space were analyzed for biofilm formation by variable pressure-scanning electron microscopy (VP-SEM), which enables the direct visualization of the implants without the need for sputter-coating. Representative VP-SEM images of the cut ends of the implants are shown (1 of 3 mice per group, with similar results). Top panels represent a low power magnification (120x) and the bottom panels show a higher magnification (600x) of the area boxed in red. Biofilm formation is readily seen from implants harvested from infected knee joints whereas only the metal surface is seen on implants from uninfected control mice.

To evaluate whether biofilm formation occurred on the implants in our mouse model, implants were harvested from euthanized mice on post-operative days 7 and 14 (Figure 5). To evaluate biofilm formation, we used variable-pressure scanning electron microscopy (VP-SEM), which allows for visualization of biologic samples in their natural state, as there is no need to coat them with a conductive film required for traditional SEM. Thus, VP-SEM enabled the visualization of biofilms on the implants without typical artifacts (dehydration, collapse, distortion, shrinkage, condensation, and aggregation) associated with conventional SEMs that require fixation and sputter coating. Mice inoculated with *S. aureus* had prominent biofilm formation on the cut end of the implants harvested on 7 and 14 post-operative days. In contrast, uninfected mice, which did not have any bacterial inoculation at the time of surgery, had no detectable biofilm formation and the visualized metallic implant surface was virtually identical to implants prior to surgery (Day 0). Thus, the bacteria infected the joint tissue (Figure 4) and also formed a biofilm on the implant, which is consistent with biofilm formation that occurs in post-arthroplasty infections in patients [8]–[11].

### A novel antibiotic-impregnated implant coating to treat *S. aureus* post-operative joint infection

**Figure 6:**
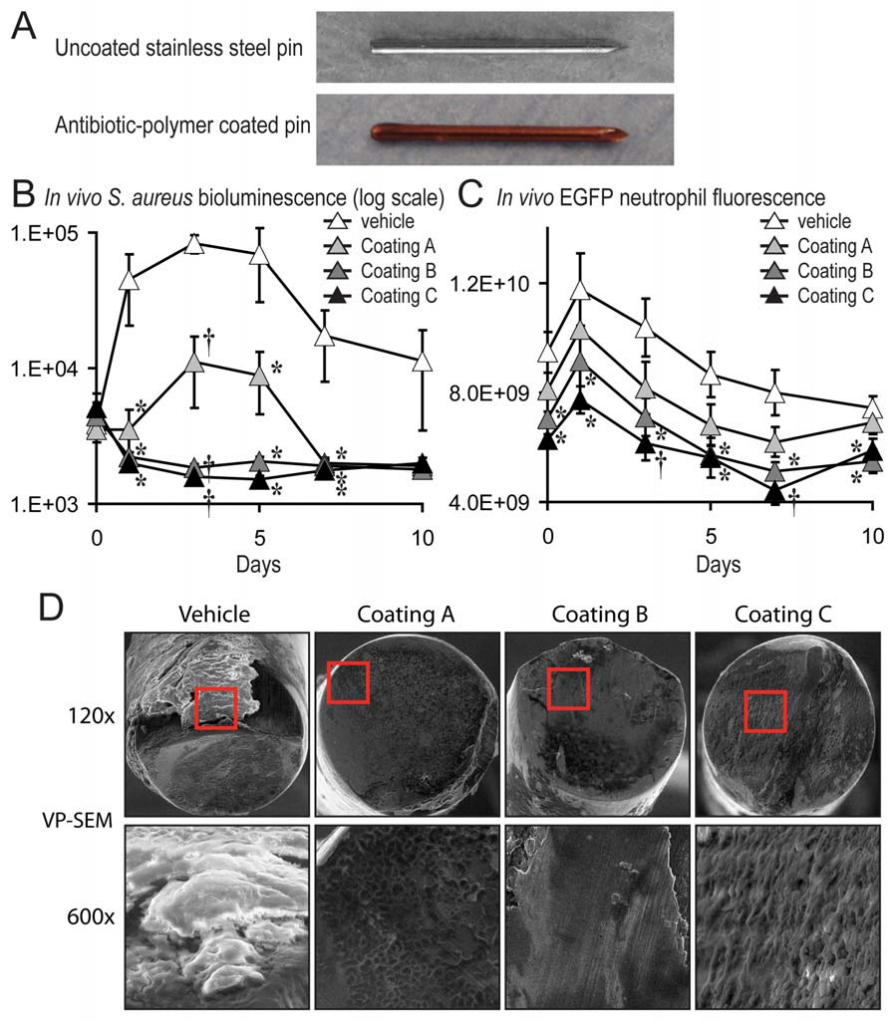
A novel antibiotic-impregnated implant coating results in reduced *S. aureus* infection, decreased inflammation and prevention of biofilm formation. Orthopaedic-grade stainless steel K-wires were machine cut and coated with increasing concentrations of a tyrosine-based biodegradable antibiotic implant coating, which contained rifampin and minocycline or vehicle alone (coating without any antibiotic). The rifampin/minocycline concentrations for each of the coatings were: Coating A: 32.5/36.1 µg/mm^3^; Coating B: 46.1/47.7 µg/mm^3^ and Coating C: 97.4/104.2 µg/mm^3^. Furthermore, Coatings A and B were the same thickness (∼40–45 µm) and would elute at the same rate whereas Coating C would elute slower because it had double the coating thickness (∼80–90 µm). These coating implants were surgically placed into the distal femur and 5×10^2^ CFUs/2 µl of *S. aureus* was inoculated into the knee joint in the area of the cut end of the implant. (A) Representative photograph of an uncoated stainless steel implant and an antibiotic-impregnated coated implant. (B) Bacterial counts as measured by *in vivo S. aureus* bioluminescence (mean maximum flux [p/s/cm^2^/sr] ± sem) (logarithmic scale) (n = 5 mice per group). (C) Neutrophil infiltration as measured *by in vivo* fluorescence (total flux [photons/sec] ± sem) (n = 5 mice per group). (D) Representative VP-SEM images of the cut ends of the implants are shown (1 of 2 mice per group, with similar results). Top panels represent a low power magnification (120x) and the bottom panels show a higher magnification (600x) of the area boxed in red. *p<0.05 †p<0.01 ‡p<0.001 coatings A, B, or C vs. vehicle coating (Student's t-test).

This mouse model was employed to determine the efficacy of a bioresorbable polymer impregnated with rifampin and minocycline in preventing the development of an infection in the joint. This polymer was modified from a similar coating FDA-approved to prevent infections of pacemakers and implantable cardioverter defibrillators (Pivit™ AB and AigisRx™ CRM) [52]. Stainless steel K-wires were coated by three coating formulations (Coatings A, B and C), which contained increasing concentrations of the antibiotics, and one vehicle control coating (no antibiotic) (Figure 6A). In addition, Coatings A and B had the same thickness and would elute the antibiotics at a similar rate whereas Coating C was double the thickness and would elute slower.

These antibiotic-coated implants were surgically placed into the distal femurs of LysEGFP mice and the knee joint space was inoculated with 5×10^2^ CFUs of *S. aureus. In vivo* imaging was performed on post-operative days 0, 1, 3, 5, 7 and 10 as in Figure 3. Coatings B and C resulted in bioluminescence signals that were highest at the time of inoculation and were reduced to background levels by day 3 (Figure 6B). Coating A resulted in bioluminescence signals that were less than the vehicle alone but did increase between 0–3 days before decreasing to background levels by day 7. As expected, the vehicle control coating, which contained no antibiotics, did not inhibit bacterial growth and resulted in bioluminescent signals that were up to 20-fold higher than the initial inoculum and up to 50-fold higher than the two most effective antibiotic-impregnated implant coatings (Coatings B and C). Thus, the antibiotic-impregnated coatings substantially reduced the bacterial burden and prevented infection in post-operative joints as measured by *in vivo* bioluminescence imaging. Since Coating A resulted in some bacterial growth, whereas no growth was detected with Coatings B or C, it is likely that both the drug concentration and elution rate contributed to the efficacy of these coatings.

The antibiotic-eluting coated implants also substantially reduced clinical signs of inflammation. Mice with Coatings B and C ambulated with notably less guarding of the operative leg than mice with vehicle-coated implants. To obtain a quantifiable measurement of the infection-induced inflammatory response, *in vivo* fluorescence of EGFP-neutrophils was measured in these LysEGFP mice (Figure 6C). Coatings B and C, which were most effective in reducing bacterial burden, had EGFP-neutrophil fluorescent signals that were reduced to background levels (i.e. no detectable inflammation) by post-operative day 5. These data demonstrate that antibiotic-impregnated implant coatings markedly reduced the infection-induced neutrophil recruitment and inflammation in a concentration- and elution-dependent fashion.

To determine whether the antibiotic-impregnated implant coatings had any impact on biofilm formation, the implants were harvested from mice on post-operative day 7 and biofilm formation was evaluated by VP-SEM (Figure 6D). All three antibiotic-impregnated implant coatings (A, B and C) prevented biofilm formation on the cut surface of the pin within the knee joint. In contrast, the vehicle coated pin had readily detectable biofilm formation.

## Discussion

Infections after total joint arthroplasty represent a clinically devastating complication [1], [2]. These infections are exceeding difficult to treat because the implanted materials provide avascular surfaces to which bacteria adhere [8]–[11] and form biofilms, which block the penetration of immune cells and antibiotics [53]–[59]. To evaluate potential preventative or therapeutic strategies against post-arthroplasty infection, we developed a mouse model of post-arthroplasty *S. aureus* infection that provides real-time noninvasive measurements of infection and inflammation. This mouse model, which uses advanced *in vivo* bioluminescence and fluorescence imaging, accurately detected a bacterial inoculum as low as 500 CFUs and noninvasively and longitudinally permitted measurement of the bacterial burden and the ensuing neutrophil inflammatory response in a live animal over the course of at least 10 post-operative days. The ability of only 500 CFUs of bacteria to induce an infection in the post-operative joints is remarkable given that an inoculum of 2×10^6^ CFUs was required in our previous work to induce an infection in mouse skin [44]–[47]. This is consistent with the notion that the immune-privileged site of the joint combined with the foreign implanted material creates an environment that is highly susceptible to bacterial infection [8]–[11]. The validity of this model was confirmed with (1) traditional CFU counts, which demonstrated that *in vivo* bioluminescence highly correlated with the numbers of CFUs adherent to the implants and within the infected bone and joint tissue and (2) histologic analysis, which demonstrated Gram-positive bacteria and neutrophil infiltration in the surrounding joint tissue. Furthermore, high resolution scanning electron microscopy techniques, VP-SEM, enabled the visualization of biofilms that formed on the metallic implants in their natural state without sputter-coating them with a conductive film. Finally, this model was successfully used to determine the efficacy of an antibiotic impregnated coating containing minocycline and rifampin, to reduce the infection, decrease inflammation and prevent biofilm formation on the implants.

This mouse model of post-arthroplasty *S. aureus* infection has unique elements that may complement or provide an alternative to other previous animal models, which were performed in larger animals, such as dogs, rabbits and rats [32]–[43]. First, this model provides longitudinal, real-time quantification of the bacterial burden and the neutrophil response in the infected joint. Unlike the previous models, which required euthanasia to determine the bacterial burden at subsequent time points (as tissue is required), this model replaces euthanasia with noninvasive real-time *in vivo* imaging. Second, previous models have presented few mechanisms for accurately quantifying the infection-induced inflammation, which is responsible for the clinical symptoms of swelling, immobility and pain. In our model, a direct measurement of the host neutrophilic inflammatory response in the infected post-operative joints was obtained by combining the use of a mouse line that possesses fluorescent neutrophils (LysEGFP mice) with advanced *in vivo* fluorescence imaging techniques [47], [48]. Additionally, as the wavelengths for bioluminescence (490 nm) and fluorescence (515–575 nm) are distinct, the bacterial burden and neutrophilic inflammatory response can be measured sequentially within the infected post-operative joints longitudinally in the same animals in real-time without the need for euthanasia. Taken together, this mouse model of post-arthroplasty infection provides longitudinal tracking of both bacterial burden and inflammatory response while substantially reducing animal usage, labor, material and experimental costs.

One limitation of the model described here is the use of the SH1000 *S. aureus* bioluminescent strain which contained the lux genes within a plasmid. Although the *in vivo* bioluminescent signals correlated with the corresponding *ex vivo* bacterial CFUs, the plasmid was only stable for the first 3 days of broth culture, such that after this time point the *in vivo* bioluminescent signals obtained using this strain may underestimate the actual bacterial burden. In addition, other factors may additionally affect the rate of plasmid loss. Future studies should be directed an improving this model, by developing a *S. aureus* clinical isolate in which the lux genes are inserted into the chromosome so that the bioluminescent signals will not be lost over time. Alternatively, there are commercially available *S. aureus* strains in which the lux plasmid is within the chromosome such as Xen29 (Caliper Life Sciences) [60]–[62].

Lastly, we evaluated whether this mouse model could be used to evaluate the efficacy of an antibiotic implant coating to reduce infection. The antibiotic-impregnated bioresorbable tyrosine-derived polymer coating, which slowly elutes minocycline and rifampin, was clinically effective in reducing bacterial load, preventing the infection, decreasing neutrophil infiltration/inflammation and preventing biofilm formation on the implants. A previous study in a rabbit intramedullary screw *S. aureus* osteomyelitis model, found that minocycline and rifampin sprayed onto the implant without an elution polymer was only partially effective in preventing colonization of the implant and infection of the bone [39]. Although our study differed in the animal model and method of bacterial inoculation, the presence of the antibiotic-impregnated bioresorbable polymer was more effective, suggesting that the elution of antibiotics may be an important factor when developing antimicrobial implant coatings. These results suggest that further study of this coating as a modality to prevent infections associated with the use of orthopaedic implants is appropriate. It should be mentioned that in addition to studying novel antimicrobial implant coatings, this model could also be used to evaluate other potential therapeutic strategies to treat biofilm-associated infections, such as antibodies targeted against biofilm factors [63].

There are some limitations of our mouse model of post-arthroplasty infection. First, we recognize that this model dramatically simplifies the steps involved in total knee arthroplasty: we did not remove the cartilage surface from the femur or tibia, no implant was placed on the tibial side and all implants were stainless steel. There may have been interplay between the cartilage and the bacteria in our model that would not occur in a human (as cartilaginous surfaces are excised during the arthroplasty) and other metals or materials used in a human arthroplasty may have different susceptibilities to bacterial infection. Additionally, the model and the antibiotic coating were used only to study infections that were acquired during the surgical procedure and not against late infections, such as those that occur through hematogenous spread. Finally, it is clear that the elution properties of the antibiotic from the alloys used in real implants would have to be assessed before evaluating the efficacy of this strategy in human patients.

Despite these weaknesses, we believe that the histologic, bacterial cell counts and scanning electron microscopic data confirms that our model depicts the *in vivo* behavior of bacteria on and around metal implant in the setting of a post-surgical joint. While all small animal models inherently simplify the surgical procedure they aim to simulate, this model is unique in that it provides real-time longitudinal tracking of infection and inflammation in a post-surgical joint.

Taken together, this mouse model may serve as an alternative noninvasive, cost-effective and accurate *in vivo* representative model of a post-arthroplasty *S. aureus* infection. This model could be potentially be used to provide important information about *in vivo* clinical efficacy of preclinical preventative or therapeutic modalities against post-arthroplasty infections before more extensive studies in larger animals and in human subjects.
